# Effect of Polymers and Storage Relative Humidity on Amorphous Rebamipide and Its Solid Dispersion Transformation: Multiple Spectra Chemometrics of Powder X-Ray Diffraction and Near-Infrared Spectroscopy

**DOI:** 10.3390/ph13070147

**Published:** 2020-07-10

**Authors:** Yuta Otsuka, Yuiko Utsunomiya, Daiki Umeda, Etsuo Yonemochi, Yayoi Kawano, Takehisa Hanawa

**Affiliations:** 1 Faculty of Pharmaceutical Sciences, Tokyo University of Science, 2641 Yamazaki, Noda, Chiba 278-8510, Japan; y.otsuka36156803@gmail.com (Y.O.); stitch_yuiko@yahoo.co.jp (Y.U.); y.kawano@rs.tus.ac.jp (Y.K.); 2 Research Institute for Science and Technology, Tokyo University of Science, 2641 Yamazaki, Noda, Chiba 278-8510, Japan; 3 Department of Physical Chemistry, School of Pharmacy and Pharmaceutical Sciences, Hoshi University, 2-4-41 Ebara, Shinagawa-ku, Tokyo 142-8501, Japan; d.a.i.k.i102475@gmail.com (D.U.); e-yonemochi@hoshi.ac.jp (E.Y.)

**Keywords:** rebamipide, X-ray diffraction, near-infrared spectroscopy, principal component analysis

## Abstract

This study aimed to investigate the effect of polymers and storage relative humidity on amorphous rebamipide (RB) and its solid dispersion phase transformation using chemometrics based on multiple datasets. The amorphous RB was prepared using particle mixture and grinding methods with hydroxypropyl cellulose, polyvinylpyrrolidone, and sodium dodecyl sulfate. Prepared amorphous RB and solid dispersion samples were stored under a relative humidity of 30% and 75% for four weeks. Infrared spectra of the dispersion samples suggested that the hydrogen bond network was constructed among quinolinone, carbonyl acid, and amide of RB and other polymers. The dataset combining near-infrared (NIR) spectra and powder X-ray diffractograms were applied to principal component analysis (PCA). The relationship between diffractograms and NIR spectra was evaluated using loadings and the PCA score. The multiple spectra analysis is useful for evaluating model amorphous active pharmaceutical ingredients without a standard sample.

## 1. Introduction

**Figure 1:**
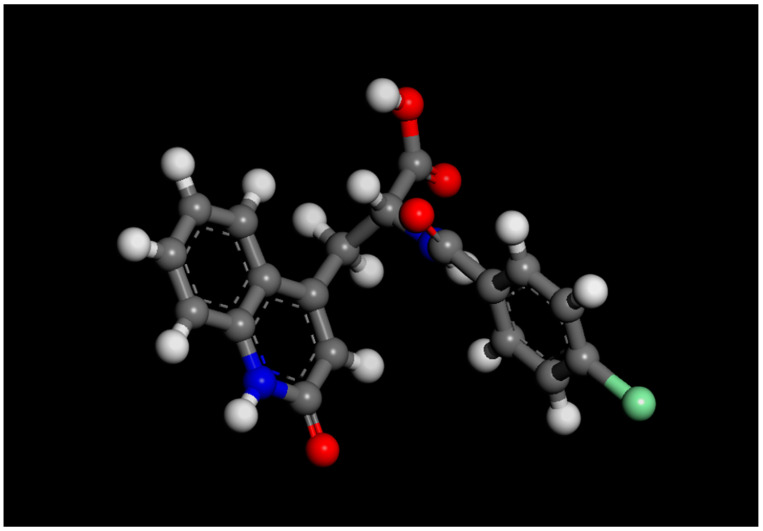
A rebamipide molecule structure.

Rebamipide (RB) is one of the treatments used for gastric ulcer, and it is known to have cytoprotective and anti-ulcer effects. Figure 1 shows a rebamipide molecule structure. It is known that the major pharmacological action of RB is the suppression of gastric mucosal damage by increasing prostaglandin E_2_ content in the gastric mucosa [1,2,3]. In addition, RB has various anti-inflammatory pharmacological actions, such as free radical inhibition, neutrophil activation inhibition [4,5,6], and inflammatory cytokine production inhibition [7,8], a mucin-like substance in the gastric mucosa [9]. Recently, it was reported [10] that anti-ulcer and cytoprotective activities of RB, such as enhancement of mucosal defense and scavenging free radicals, is effective for stomatitis treatment. In many cases, the active agents are dispersed or dissolved in the solvent, which has a mucoadhesive property. However, the solubility of RB is quite low (0.0001 or 0.013% (*w*/*v*) at pH 3 or 7, respectively); therefore, there is still room for improvement.

Dissolution behavior and water solubility of active pharmaceutical ingredients (APIs) are essential parameters for pharmaceutical preparation planning. The mechanochemical synthesis for amorphization and solid dispersion technology is one practical approach that was well investigated to improve the water solubility of APIs [11]. The solubility of amorphous and solid dispersion pharmaceuticals would improve by increasing their chemical potential; however, they are unstable in high humidity conditions [12]. Both solubility and phase transformation control are required in unstable APIs. Many studies reported the effect of polymers on amorphous solid dispersion to control API polymorphic transformation. Polyvinylpyrrolidone (PVP) [13] and hydroxypropyl cellulose (HPC) [14] are widely used in solid dispersion. Both polymers are known as water-soluble, water absorbant, and non-toxic excipients. Garcia et al., reported that mebendazole-HPC solid dispersion improves the dissolution ratio due to the decrease in API crystallinity and alters the wetting effect of HPC and crystal morphology [15]. Pongpeerapat et al, reported the effect of sodium dodecyl sulfate (SDS) concentration on particle size and their intermolecular interactions using solid-state NMR [16].

Meanwhile, controlling the quality of pharmaceutical products using thermal analysis, dissolution test, and powder X-ray analysis often requires significant time, cost, and manpower [17]. Analysis methods of products need to be quick, highly efficient, non-destructive, and non-contact. The Food and Drug Administration recommends inline and online process analysis [18] in the pharmaceutical fields that use non-contact and non-destructive analysis methods, such as near-infrared (NIR) [19], Raman [20], and THz [21] spectroscopy. The process analysis technologies of these have been accepted to control the final product quality for decades [22]. NIR spectroscopy is applied to continuous quality control processes of pharmaceutical products [23] due to its characteristics. Additionally, the handheld type NIR spectrometers are commercially available at reduced costs [24]. Silva et al., investigated mebendazole polymorphism (form A, B, and C) evaluation using a portable NIR spectrometer [25]. Three forms of mebendazole spectra show the specific peak differences at approximately 1500 nm. NIR measurement focuses on the detection of compounds with molecular vibration anharmonicity (e.g., CH, NH and OH bonds) [26]. The 1400 nm region was first known as the mode of the OH bond present in water and hydroxyl groups. However, NIR spectra of pharmaceutical products with multiple compounds were intrinsically complex and overlapping. An interpretation was difficult; therefore, chemometrics has been applied to the spectral analysis. Chemometrics studies based on NIR spectra were reported as qualitative classification [27], quantitative regression [28], predicting the physical properties of the product [29], process analysis at granulation [30], drying, granulation [31], compressed tablets [32], and many more.

Principal component analysis (PCA) is one of the unsupervised learning multivariate analysis methods. It has also been applied to pharmaceutical NIR spectra [33]; the spectra dataset is turned out into weights for each wavenumber variable, called loadings, and contains the original data in a rotated coordinate system scores matrices. Since PCA does not require an objective variable, it is useful in evaluating changes in the time characteristics of samples. In our previous chemometric study, we reported phase transformation kinetics of metastable calcium phosphate based on IR spectra using decomposed loadings and PCA score [34].

Chemometrics, based on multiple datasets, called combining spectroscopic data, have the potential to become the next generation standard method for correlation studies and/or monitoring systems in industrial processes. Lambert et al., suggested combining Raman and mid-infrared (MIR) spectra for red paint analyses [35]. Schoonjans and Massart reported a chemometric approach based on combining mass and infrared spectra to characterize 61 compounds [36]. Alamprese et al., reported minced beef evaluation based on combining UV–vis, NIR, and MIR spectra [37]. De Groot et al., improved the predicted accuracy of yarn properties based on the combination of selected MIR and Raman spectra [38].

In our previous study, we reported the effect of HPC molecular weight on RB nanoparticle crystallinity, particle size, IR shifts due to carbonyl acid intermolecular interactions, zeta-potential and dissolution behaviors [39]. Additionally, we also reported chemometric studies on pseudo-polymorphism of API using attenuated total reflection (ATR)-MIR spectra with MCR-ALS [40] and Raman spectra with partial least squares (PLS) correlation [41]. In this study, we investigated the effect of polymers and storage relative humidity on amorphous RB transformation by chemometrics based on NIR spectra and powder X-ray diffractograms (PXRD) patterns multiple datasets to find an evaluation method of recrystallization on amorphous RB.

## 2. Results and Discussion

### 2.1. Effects of High Humidity Storage on Amorphous RB and Bulk RB Samples

**Figure 2:**
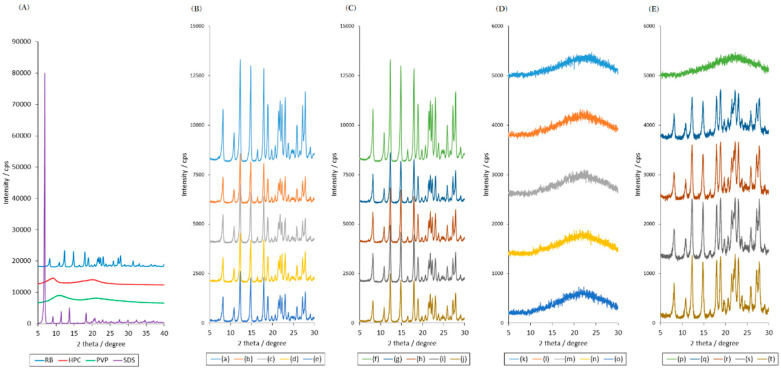
PXRD pattern of bulk materials (**A**) and stored samples (**B**–**E**). RB form I PM sample stored in RH 30% (**B**) and RH 75% (**C**). Amorphous RB sample stored in RH 30% (**D**) and RH 75% (**E**). Storage time: 0 weeks (a, f, k, and p); 1 week (b, g, l, and q); 2 weeks (c, h, m, and r); 3 weeks (d, i, n, and s); 4 weeks (e, j, o, and t). RH; relative humidity. RB; rebamipide. PXRD; powder X-ray diffractograms.

Figure 2 shows the PXRD patterns of stored samples, Amorphous RB, and bulk RB. Figure 2A shows RB form I, HPC, PVP and SDS patterns. HPC and PVP show broad two peaks. SDS have strong diffraction peak at 6.8 degree. Figure S1 shows the images of bulk materials and stored Amorphous RB, bulk RB and associated samples in controlled humidity desiccators. The bulk material samples of PVP in relative humidity (RH) 30% and RH 75% were transformed into hard grassy samples under controlled humidity conditions. All of the stored RB samples were powdery and fluid. It was reported that there are several RB pseudo-polymorphisms (i.e., monohydrate) and RB form II [42,43]. In the case of bulk RB storage at low (Figure 2B) and high humidity (Figure 2C), there was no pseudo-polymorphic or polymorphic transformation in the stored bulk RB sample, indicated by no change in the diffractogram pattern at RH 30% and RH 75% for four weeks. However, after four weeks of RH 30% and 75% storage, the intensity of the unique RB peak at 8.20° decreased respectively to 54.5% and 60.4%. Additionally, the diffractogram baseline of both condition samples decreased. Decreasing PXRD baseline intensities of amorphous RB also indicated the decreased density of the sample. The amorphous RB samples stored at RH 30% (Figure 2D) showed broad diffractograms in four weeks. Conversely, amorphous RB stored in the RH 75% desiccator (Figure 2E) showed RB unique diffraction pattern in one week. The RB pattern intensity increased from week 1 to week 4. The recrystallization of amorphous RB samples due to high humidity was indicated. The amorphous RB samples (Figure 2D,E) showed a broad diffractogram due to non-periodic forms in the sample, indicating that bulk RB was transformed into amorphous form by ball milling.

**Figure 3:**
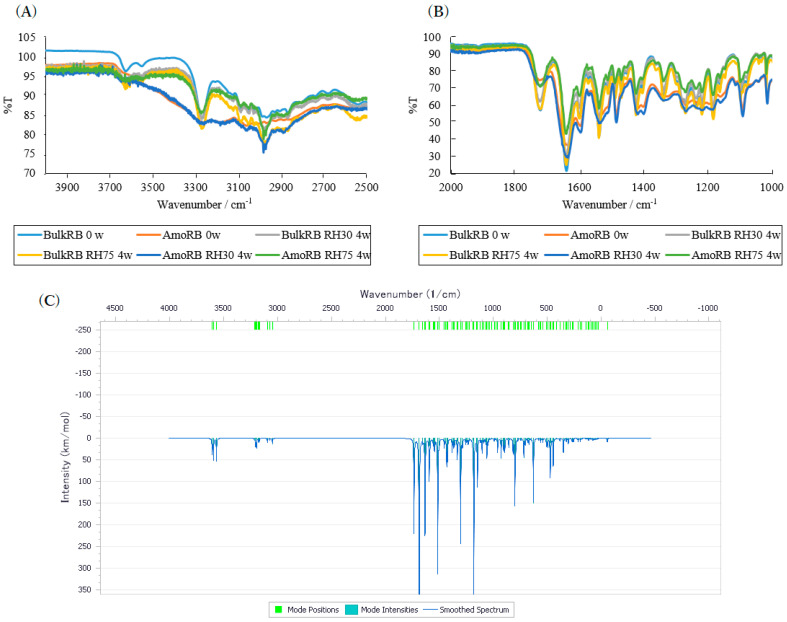
Attenuated total reflection-mid-infrared spectra of RB form I, ground RB and stored samples. RB form I sample stored for 4 weeks in RH 30% and RH 75%. The two regions of 4000 to 2500 cm^−1^ (**A**) and 2000 to 1000 cm^−1^ (**B**). Simulated IR spectra of RB molecule based on ab initio calculation (**C**). RH; relative humidity. RB; rebamipide.

Figure 3 shows obtained MIR spectra of stored samples, ground RB, and bulk RB. Effect of amorphization on IR spectra was indicated by spectra broadening in the region of 3700 to 2500 cm^−1^ and 2000 to 1000 cm^−1^. Molecular orbital calculation of an RB molecule in vacuum was simulated to clear the RB vibration modes. The B3LYP function was performed for frequency analysis with geometrical optimization. Figure 3C shows IR vibration analysis of the RB molecule based on B3LYP in vacuum condition. The final optimized total energy of B3LYP was calculated in 1687 Hartree, and the results are listed in Table S1. According to the simulation of B3LYP, the IR vibration modes of 1720, 1640, and 1632 cm^−1^ indicated carboxylic acid C=O (Figure S3c), quinolinone C=O (Figure S3b), and amide C=O (Figure S3a) stretch, respectively.

MIR spectra of amorphous RB and stored amorphous RB at RH 30% showed broad peaks at NH stretch next to the carboxyl group, OH stretch of the carboxyl group and NH stretch of quinolinone in the region from 3700 to 2900 cm^−1^. The pronounced peak at 3272 cm^−1^ was due to cyclic CH stretch of quinolinone. Effect of amorphization on RB was indicated by the carboxyl C=O stretch of 1726 cm^−1^ and broad peaks. The carboxyl acid C=O and quinolinone C=O stretch are specific interaction points of hydrogen bond networks in the RB crystal. The spectra broadening suggested that the hydrogen bond network of amorphous RB was different from that of crystalline RB. The spectra correlation of form I and amorphous form were calculated to evaluate spectra patterns of stored samples. The spectra correlation of started material samples and stored samples are listed in Table 1. Both correlations of RB form I samples stored in RH 30% and RH 75% were over 0.99. Based on the calculated *R*^2^ values of spectra, correlation without sample destruction was suggested. Comparing the absorption intensity of amorphous RB form I, it was indicated that it decreased with amorphization.

**Figure 4:**
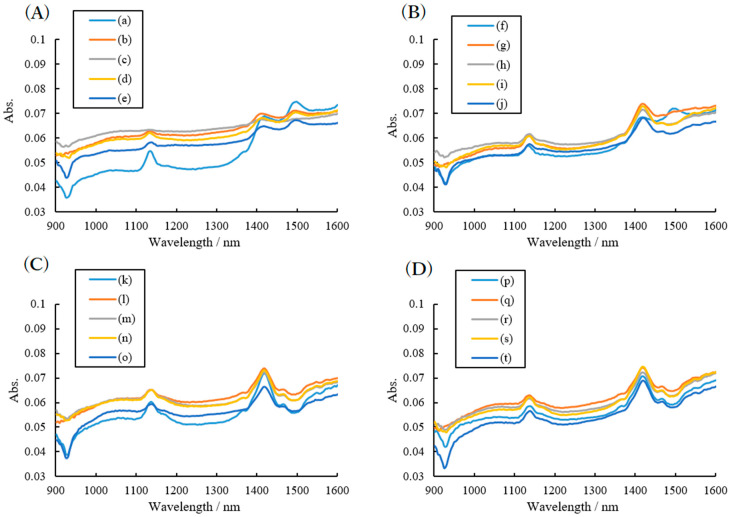
Near-infrared spectra of stored samples. Ground RB sample stored in RH 30% (**A**) and RH 75% (**B**). Bulk RB sample stored in RH 30% (**C**) and RH 75% (**D**). Storage time: 0 weeks (a, f, k, and p); 1 week (b, g, l, and q); 2 weeks (c, h, m, and r); 3 weeks (d, i, n, and s); 4 weeks (e, j, o, and t). RH; relative humidity. RB; rebamipide.

Figure 4 shows the NIR spectra of the bulk RB and prepared samples with standard normal variate (SNV) normalization. SNV normalization is one of chemometric normalize techniques widely used in NIR spectra study. After the normalization, each spectrum will have a mean of 0 and a standard deviation of 1. An RB molecule has combined structures of quinolinone, carboxyl acid, and chlorobenzene. Intense absorption bands were observed around 1140 and 1422 nm in the bulk RB sample. Additionally, as observed from the spectra, there was an apparent difference in the 1498 nm peaks of amorphous RB samples. The NIR spectra of the amorphous sample stored in RH 30% showed a 1498 nm peak at four weeks; however, the peak intensity was decreased. Compared to RH 30%, amorphous RB stored in RH 75% showed crystallinity patterns detected using PXRD, and the amorphous specific peak disappeared after recrystallization. From these results, it was found that qualitative analysis of polymorphism was possible with both MIR and NIR spectra. Three analytical methods indicated amorphous RB recrystallized in RH 75% storage, and it was stable in RH 30% storage conditions. In the case of amorphous RB prepared by the grinding method, RB was stable in crystal form in RH 75%.

Takeuchi et al., reported moisture-induced recrystallization in amorphous lactose at high humidity [44]. Mahlin et al., reported amorphous lactose transformation kinetics using thermal activity monitor, PXRD, and AFM analysis [45]. The amorphization is one of the strategies to improve water solubility; however, keeping amorphous molecules at high humidity is also important. Lust et al., reported that polyvinyl caprolactam-polyvinyl acetate-polyethylene-glycol graft copolymer can be applied to prevent piroxicam recrystallization [46]. In the next section, polymer excipients were applied to control RB phase transformation.

### 2.2. Effects of High Humidity Storage on the Mixture of RB Sample, SDS and PVP

Co-grinding RB with SDS and PVP (RSP) was performed at high humidity to enhance recrystallization of amorphous RB to mimic water’s plasticization effect. The effect of polymers on co-grinding RSP samples and water’s plasticization effect was investigated using a humidity-controlled desiccator. The mixture sample RSP at a mass ratio of 1:1:5 (RB:SDS:PVP) was prepared. RSP stored in RH 30%, or RH 75% were analyzed in the same way as in the previous section. Figure S2 shows images of physical mixture (PM) and ground mixture (GM) of RSP samples stored in RH 30% and RH 75%. The prepared samples were powdery and fluid. Both PM and GM samples were transformed into a hard, glassy sample after one week in RH 75%. The RSP PM and GM samples stored in RH 30% were powdery and fluid after four weeks.

**Figure 5:**
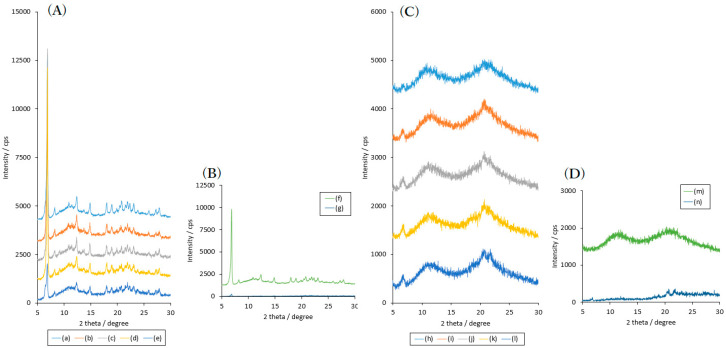
PXRD spectra of stored RB, SDS, and PVP mixture samples. Amorphous RB sample stored in RH 30% (**A**) and RH 75% (**B**). Bulk RB sample stored in RH 30% (**C**) and RH 75% (**D**). Storage time: 0 weeks (a, f, h, and m); 1 week (b, g, i, and n); 2 weeks (c and j); 3 weeks (c and k); 4 weeks (e and l). RH; relative humidity. RB; rebamipide. SDS; sodium dodecyl sulfate. PVP; polyvinylpyrrolidone.

Figure 5 shows the powder X-ray diffractograms of the RSP samples. Prepared PM RSP sample showed SDS-specific diffraction peaks at 6.80° and unique diffraction patterns of RB form I on bulk PVP diffractograms. Samples stored in RH 30% (Figure 5A) had a decrease in the 6.80° SDS peak and the RB form I patterns were still present after four weeks. The sample diffractograms indicated that RB in PM RSP samples had crystallinity, and it was stable in the RH 30% condition. On the other hand, PM RSP diffractograms stored in RH 75% sample (Figure 5B) showed SDS, PVP, and RB form I patterns at zero weeks; however, were transformed into almost non-periodic diffractograms with a small SDS peak after one week (Figure 5B, line g). The diffractograms indicated that PM RSP samples had a driving force of self-glass transformation in RH 75%. Prepared RSP GM samples (Figure 5C) showed two broad peaks in the ranges of 10° to 15° and 17° to 23°. The broad diffractograms of the sample were similar to bulk PVP diffractograms. Van den Mooter et al., reported that PXRD patterns of temazepam-PVP K30 (4:6) solid dispersion, prepared by co-evaporation, shows bulk PVP K30 diffraction patterns [47]. The diffractogram of our RSP GM sample indicated that under milling stress, the solid was at least mostly amorphized, but the fact that recrystallization was observed at 75% RH showed that the amorphous state was not stable. Furthermore, the 30% RH samples of diffractograms showed a tiny evolution at 30% RH; however, a peak around 6 to 7° due to SDS diffractograms appearing at four weeks. There was at least a suspicion of two peaks around 21 to 22°, at the same position after storage in 75% RH. The PXRD patterns after four weeks in RH 30% still indicated an amorphous pattern. After storing the sample in RH 75% for one week, there were small diffraction peaks at 21° and 22° instead of the bulk PVP diffractogram. After two weeks, samples were utterly transformed into a glassy solid sample.

**Figure 6:**
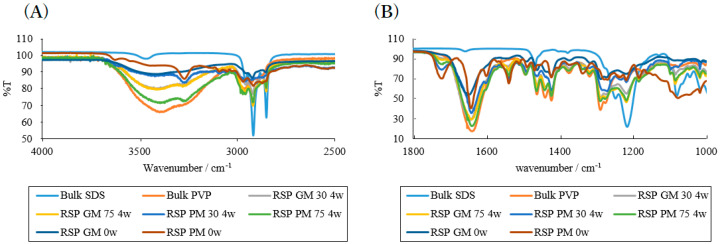
Attenuated total reflection-mid-infrared spectra of bulk SDS, bulk PVP and stored RSP samples. RSP samples, bulk SDS and bulk PVP samples were stored in RH 30% and RH 75%. Bulk RB sample was stored for 4 weeks in RH 30% and RH 75%. The two regions of 4000 to 2500 cm^−1^ (**A**) and 1800 to 1000 cm^−1^ (**B**). RH; relative humidity. RB; rebamipide. SDS; sodium dodecyl sulfate. PVP; polyvinylpyrrolidone. w; weeks.

ATR-MIR spectra of the 4-week-stored and bulk samples are shown in Figure 6. The peak shift evaluation due to the molecular interaction of the co-grind mixture samples, such as polymer and API, is one of the conventional methods for amorphous analysis. It is known that SDS show pronounced carbon chain IR peak due to CH_2_ stretch and asymmetric stretch at 3000 to 2800 cm^−1^. IR spectra of bulk SDS and stored RSP samples had several peaks. Bulk PVP had broad peaks in the range of 3700 to 3100 cm^−1^ due to OH stretching region [48]. PVP is known for its water absorption ability; therefore, the region included the vibration of the water absorption on PVP [49]. The peak at 1681 cm^−1^ was due to the C=O stretch. Crowley and Zografi reported the IR peak shifts of indapamide, ursodeoxycholic acid or indomethacin-PVP solid amorphous peak assignments and their glass transformation physicochemical properties [50]. The RB peaks at 3627 cm^−1^ and 3546 cm^−1^, due to the NH stretch of quinolinone and amide NH stretch of the carboxylic acid, disappeared when GM was prepared and stored. In contrast, cyclic CH stretch of quinolinone at 3262 cm^−1^ was shown in stored samples. The peak shifted from 3272 cm^−1^ in form I due to amorphization. Additionally, the peak of carboxyl acid C=O stretch vibration had increased transmittance after storage. These disappeared peaks agreed with their crystallinity from PXRD patterns, and essential points of hydrogen bonding were reported in other studies of RB pseudo-polymorphism [50,51]. The disappearance suggested that carboxylic acid, the amide of quinolinone and the amide of carboxylic acid construct hydrogen bond networks with PVP.

**Figure 7:**
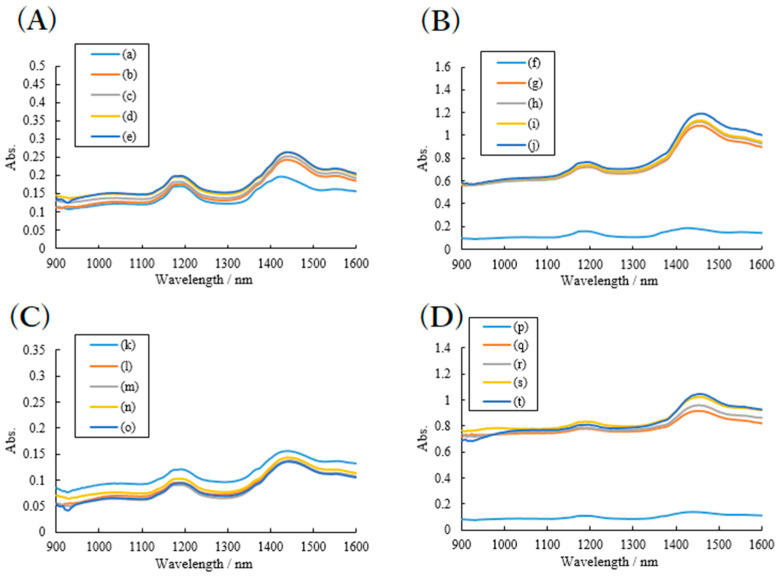
SNV normalized near-infrared spectra of stored RB, SDS, and PVP samples. Physical mixture samples in RH 30% (**A**) and RH 75% (**B**). Ground mixture samples in RH 30% (**C**) and RH 75% (**D**) ranging from 900 to 1600 nm. Storage time: 0 weeks (a, f, k, and p); 1 week (b, g, l, and q); 2 weeks (c, h, m, and r); 3 weeks (d, i, n, and s); 4 weeks (e, j, o, and t). RH; relative humidity. RB; rebamipide. SDS; sodium dodecyl sulfate. PVP; polyvinylpyrrolidone.

Figure 7 shows the NIR spectra of the prepared sample and bulk materials. The spectra had considerable overlapped absorbance peaks. From 1140 to 1250 nm, peaks suggested the second overtone of CH_2_ and CH_3_ in SDS and PVP. From 1350 to 1500 nm, peaks indicated the mixture of SDS and PVP and water peaks. The 1140 to 1250 nm peak decreased in the samples stored in RH 75% for four weeks. The decrease in the absorbance agreed with the glass transformation of the PM and RSP GM samples. For PM RSP samples stored in RH 30%, the NIR spectra shifted from 1414 to 1452 nm due to the decrease in SDS crystallinity, and water absorption was suggested. For PM and GM samples stored in RH 75%, the peaks at 1140 to 1250 nm broadened with glass transformation. Kreft et al., reported that chemometrics study on PVP grade is discriminated by NIR diffuse reflectance techniques [52]. They discuss that NIR spectroscopy is less suitable for direct detailed qualitative analysis than IR spectroscopy, although using chemometrics enables analytical information. NIR spectra shifts of stored RSP samples suggested the use of qualitative analysis for glass transformation.

Although it is known as a hydrophilic polymer and a water-absorbing material, this study revealed that a mixed sample of both particle mixture and grinding amorphization transformed the sample into a hard glass in two weeks in RH 75%. In another investigation, Karavas et al., reported solid dispersion systems of felodipine-PVP [53]. They found that the samples construct hydrogen bonds between felodipine and PVP and are stabilized at 40 in RH 75%. The hydrogen bond network construction in RSP samples was indicated by IR spectra evaluation. Log P of both felodipine and RB are known as approximately 4; the effect of humidity depends on physical properties of API molecules. In the case of flunarizine-PVP solid dispersions [13] prepared by a methanol solution method, PVP prevents flunarizine recrystallization. In our RSP sample, PVP prevented recrystallization. The results of hard glassy transformation indicated that it was not suitable for storage for a long time in the case of RB. However, the PVP solid dispersion has good solubility; therefore, it was effectively administered without sample transformation immediately after synthesis. Next, HPC was selected as a prevention material to inhibit recrystallization and glass transformation.

### 2.3. Effects of High Humidity Storage on RB Mixture Sample with SDS and HPC

**Figure 8:**
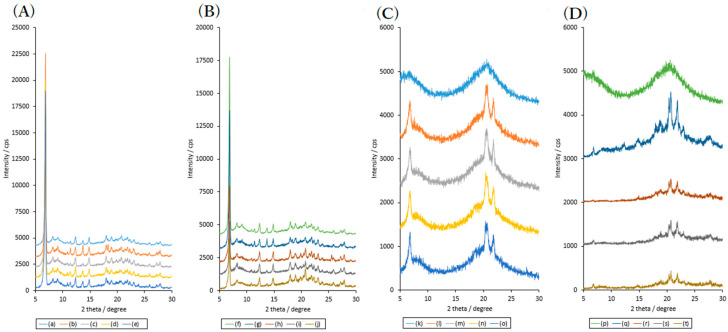
PXRD patterns of stored RB, SDS and HPC-SSL samples. Physical mixture samples under RH 30% (**A**) and RH 75% (**B**), ground mixture samples under RH 30% (**C**) and RH 75% (**D**). Storage time: 0 weeks (a, f, h, and m); 1 week (b, g, i, and n); 2 weeks (c and j); 3 weeks (c and k); 4 weeks (e and l). RH; relative humidity. RB; rebamipide. SDS; sodium dodecyl sulfate. HPC-SSL; SSL grade hydroxypropyl cellulose. PXRD; powder X-ray diffractograms.

Figure 8 shows the PXRD patterns of prepared mixture of RB, SDS, and HPC-SSL (RSH) samples. Both PM samples stored in RH 30% (Figure 8A) and RH 75% (Figure 8B) showed RB form I after four-week-storage. The intensity of the RSH sample stored in RH 75% at 6.9° (Figure 8B) decreased with storage time. The decrease indicated that SDS crystallinity in the sample was decreased by water vapor. For the GM sample (Figure 8C,D), the diffractogram of the zero-week samples was in accordance with bulk HPC diffractogram; therefore, the GM samples indicated partial amorphous solid dispersion and suggested that RB was under solid dispersion limit same as RSP samples. The stored GM RSH sample in RH 30% (Figure 8C) showed peaks at 6.9°, 20.8° and 22.4° on HPC broad peaks. The GM RSH sample stored in RH 75% (Figure 8D) no broad HPC peaks, and the previous recrystallization appeared. The broad HPC peaks indicated transformation to other phases with recrystallization. Figure S3 shows images of RSH samples stored in RH 30% and RH 75% prepared by PM and GM. All prepared RSH samples were powdery and fluid. Photographic images of the samples were shown in Supplementary Figure S4.

**Figure 9:**
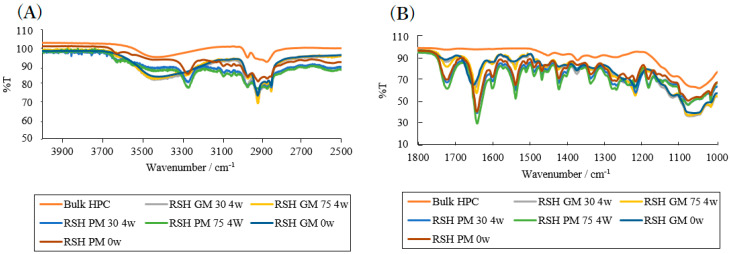
Attenuated total reflection-mid-infrared spectra of bulk HPC and stored RB, SDS, and HPC-SSL (RSH) samples. RSH samples were stored in RH 30% and RH 75%. RB, SDS, and PVP samples stored for 4 weeks in RH 30% and RH 75%. The two regions of 4000 to 2500 cm^−1^ (**A**) and 1800 to 1000 cm^−1^ (**B**). RH; relative humidity. RB; rebamipide. SDS; sodium dodecyl sulfate. HPC-SSL; SSL grade hydroxypropyl cellulose. PVP; polyvinylpyrrolidone. w; weeks.

MIR spectra of the samples were evaluated, shown in Figure 9, to clear their transformation. The IR spectra of RSH samples were obtained at 0 and 4 weeks of storage period. The spectra of PM RSH samples at week 0 and 4 showed specific RB peaks at 3624, 3627, 1722, and 1642 nm. The results agreed with the PXRD patterns that RB form I samples showed. There were no phase transformations indicated in the PM RSH sample. The spectrum of GM RSH at week 0 showed broad peaks from 3650 to 3000 cm^−1^. IR spectra of RHS GM stored in RH 30% and RH 75% still had similar peaks ranging from 3300 to 3700 cm^−1^; however, the peak at 1650 cm^−1^ shifted to 1648 cm^−1^ in RH 30%, and 1644 cm^−1^ in RH 75%. Additionally, the peak of 1727 cm^−1^ was decreased. According to the recrystallization of the PXRD pattern, it is indicated that storage in high humidity affected the intermolecular interaction transformation of HPC. The stored GM RSH samples had no peaks at 3272 cm^−1^. The results suggested that the RB molecule was fully dispersed in HPC and SDS solid departure matrix in GM RSH samples. GM RH 75% samples indicated hydrogen bond network transformation.

**Figure 10:**
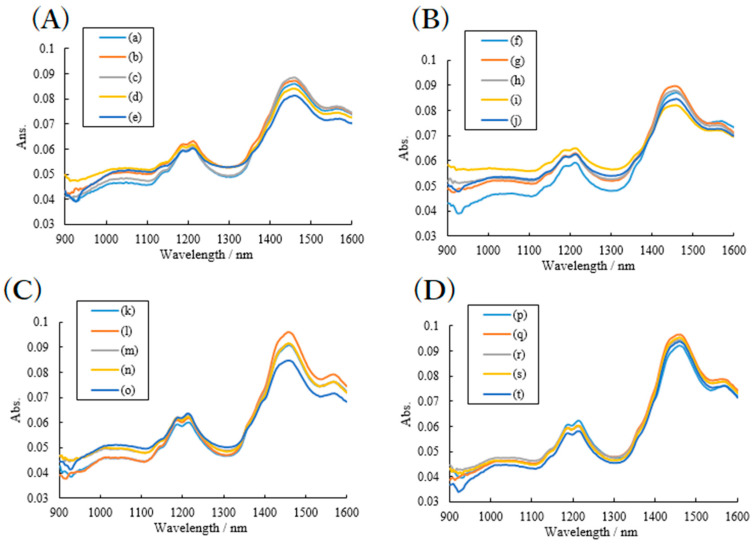
Near-infrared spectra of stored samples. Ground mixture RB, SDS and HPC-SSL (RSH) sample stored in RH 30% (**A**) and RH 75% (**B**). Physical mixture RSH samples stored in RH 30% (**C**) and RH 75% (**D**). Storage time: 0 weeks (a, f, k, and p); 1 week (b, g, l, and q); 2 weeks (c, h, m, and r); 3 weeks (d, i, n, and s); 4 weeks (e, j, o, and t). RH; relative humidity. RB; rebamipide. SDS; sodium dodecyl sulfate. HPC-SSL; SSL grade hydroxypropyl cellulose.

The time course of NIR spectra for the stored and prepared RSH samples are shown in Figure 10. NIR spectra of RH 75% samples were constant. The peak at 1400 to 1500 nm for the stored RH 30% samples increased. The spectra of the GM samples showed baseline shifts between 1250 to 1350 nm. The mechanism of recrystallization from solid dispersion is still not fully understood; however, it is acceptable that there are nucleation and crystal growth stages similar to the supersaturated solutions. Otsuka et al., reported the effect of HPC on phase transformation of carbamazepine anhydrate in a supersaturated solution [40]. Carbamazepine transformation concludes the HPC adsorption on the seed crystal surface and decreases their crystal growth. There are many reports on cellulose derivative solid dispersants (e.g., HPC, hydroxypropylmethylcellulose and hypromellose acetate succinate) [54] and surfactants [55] in the supersaturated dissolution test solution. NIR and MIR spectra and PXRD patterns of RSH samples suggested that RB recrystallization was inhibited by adsorption of HPC and SDS via the driving force of dispersed RB. Yoo et al., reported that the effect of anti-plasticization depends on API physical parameters and cannot be represented by one or two physicochemical properties; therefore, more complicated model systems based on various evaluation systems should be considered [56]. Roscigno et al., reported that the interaction takes place through both electrostatic and hydrophobic contributions between SDS and PVP by 2D NMR measurement [57]. Pongpeerapat et al., reported the intermolecular interactions in Probulcol-PVP-SDS solid dispersion sample using solid-state NMR spectroscopy [16]. The RB, SDS, and PVP interactions in solid dispersion were also indicated due to hydrogen bond networks construction, the electrostatic force between PVP-SDS and hydrophobic interactions.

### 2.4. Dynamic Vapor Sorption (DVS) of Solid Dispersion Samples

**Figure 11:**
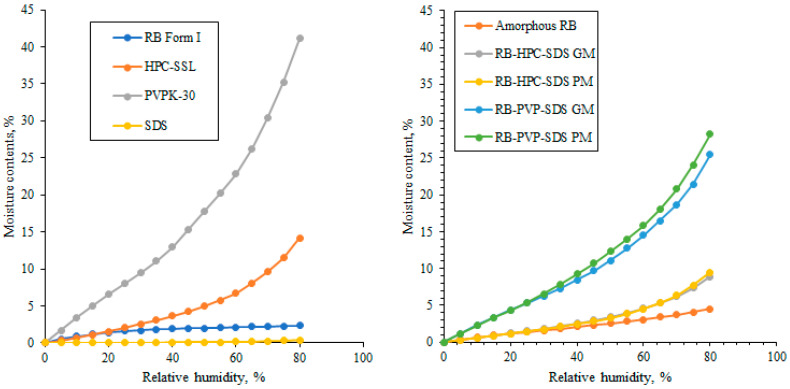
DVS results of bulk materials (**left**) and prepared samples (**right**).

Figure 11 shows DVS isotherm plots for bulk materials and prepared samples at 25 °C. Since the water content of RB form I was 2.3% at RH 80%, it was suggested that water absorption was not high. Moisture contents of amorphous RB samples were 4.5%. Bulk SDS was under 0.4%, which indicated that the water absorption ability was low. Moisture content percentage of HPC and PVP at RH 80% were 14.2% and 41.1%, respectively. At all levels of humidity, the percentage of absorbed water by HPC-based RB solid dispersion samples was consistently lower than that of PVP solid dispersion samples. At RH 80%, the integrated value-based weight ratio of the three contents and the amount of absorbed water agreed with the data of the three-component solid dispersion. The results suggested that hydrogen bond network transformation in solid dispersion samples affected sample properties of powder surface and water absorption dynamics.

**Scheme 1:**
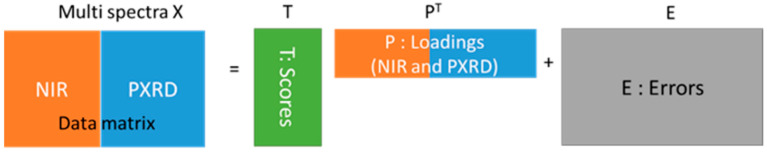
Principal component analysis for combined near-infrared-PXRD spectra dataset.

To investigate sample crystallinity and their phase transformations, Multiple dataset analysis with PCA was demonstrated. Multiple NIR-PXRD datasets can be represented in n-dimensional space, and it is difficult to comprehend the data in more dimensions (Scheme 1). It is, therefore, useful to reduce the dimensionality of measurement data [58]. Multivariate analysis methods, such as PCA, can reduce the dataset dimensionality while retaining most of the original dataset in the absorbance and diffraction.

**Figure 12:**
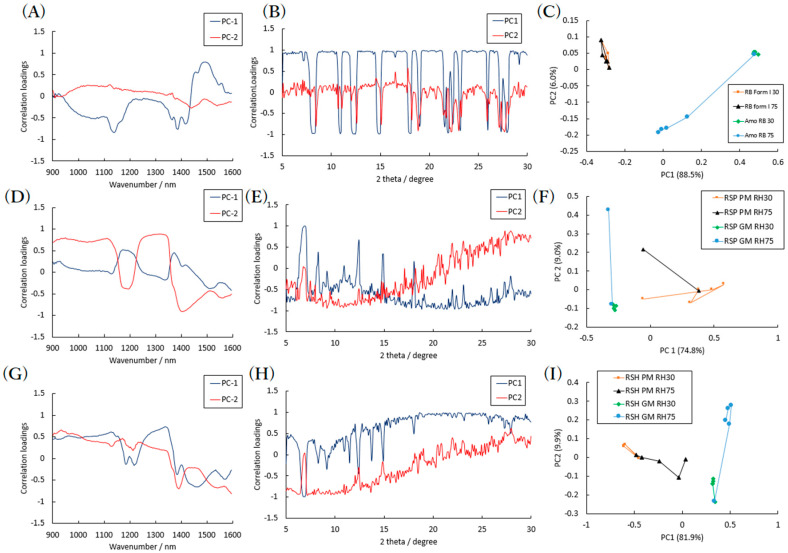
Constructed principal component analysis models based on near-infrared (NIR)-PXRD multi-dataset. NIR (**A**) and PXRD (**B**) loadings and scores (**C**) based on amorphous RB and RB form I dataset. NIR (**D**) and PXRD (**E**) loadings and scores (**F**) based on RB, SDS and PVP samples. NIR (**G**) and PXRD (**H**) loadings and scores (**I**) based on RB, SDS and HPC-SSL samples. RB; rebamipide. SDS; sodium dodecyl sulfate. HPC-SSL; SSL grade hydroxypropyl cellulose. PVP; polyvinylpyrrolidone. PXRD; powder X-ray diffractograms.

Three PCA models based on amorphous and bulk RB, RSP, and RSH samples were constructed with vector normalization to evaluate the sample crystallinity and variation from NIR-PXRD data sets. The normalized multi-spectra were applied to PCA. The cumulative percentage variance of the three models is listed in Table 2. All cumulative percentage variances of constructed models were over 83% at PC2 validation; therefore, the loadings and scores were evaluated at PC1 and PC2. The variable high loading indicated their collinear relationship. Figure 12A–C show loadings of NIR spectra and PXRD patterns, and their scores on amorphous RB and bulk RB spectra, respectively. The loading PC1 indicated a high correlation at 1150 nm and 1505 nm peaks and RB form I diffraction peaks. Scores for PC1 vs PC2 indicated three clusters on the plot. The first cluster was a low PC1 value with a high PC2 value due to crystallized RB samples. The second one was a high PC1 with a high PC2 due to amorphous RB samples. The third cluster was a recrystallized sample in the middle of PC1 with PC2. Time course of amorphous RB sample scores stored in RH 75% showed phase transformation from amorphous RB to recrystallized RB form with low crystallinity. This multispectral PCA with normalization and combination made it easier to evaluate the corresponding points. In the PXRD loading of PC2, the peak at 2θ showed high values. The samples that had crystallinity showed a peak area, and the peaks depend on the peak width and tail of peak [59]. PC2 showed the relationships between NIR spectra and PXRD tails on crystallinity. Figure 12D–F shows the loadings of NIR spectra and PXRD, and the relevant scores of RSP samples. High correlation points for PC1 were the peaks at the range of 1158 to 1202 nm, 6.8° of SDS diffraction and patterns of RB form I. The correlation regions that were approximate to 0.8 for PC2 in NIR spectra were 900 to 1100 nm and 1240 to 1370 nm. The NIR region suggested baseline shifts of the sample from a hard-glassy transformation due to a decrease in density, indicated by score variation of RSP GM RH 75% from low to high. Figure 12G–I also shows the NIR-PXRD data set loadings and scores on the stored sample of RSH. Twin peaks of 1188 and 1220 nm agreed with HPC NIR patterns shown in the PC1 loading. Loading of PC1 in the PXRD region showed SDS diffraction and RB form I pattern. PC2 score of RSH GM samples stored in RH 75% decreased from week 0 to 1. In contrast, the RSH PM and GM RH 30% samples had constant values. The PC2 score variance suggested it was due to the PXRD patterns baseline decrease, and they were shown in the variance of NIR spectra peak shifts at 1408 nm.

PCA based on NIR-PXRD patterns enabled clear complicated correlations between PXRD patterns and NIR via loading analysis. It was suggested that NIR spectrometer could perform a similar characterization as PXRD. The mobile NIR spectrometer showed the potential of evaluating the degree of amorphization with supervised learning models, such as PCR, PLS or support vector machine. The mobile NIR spectrometer is non-destructive, measurable in a glass bottle, requires less amount sample, provides quick measurement and takes up less volume [24]. The results suggested that quality control of amorphous, solid dispersions and/or polymorphism evaluation of in-hospital formulation will be possible to for pharmacists, who have the skill of supervised learning with open source code language. The PCA results suggested that the NIR spectrum could be expressed as a linear map of PXRD patterns of the solid dispersion samples. Solid dispersion samples that could not be obtained with a single measurement data could be evaluated using multispectral PCA.

## 3. Materials and Methods

### 3.1. Materials

RB (Tokyo Chemical Industry Co., Ltd., Tokyo, Japan) and SDS (Fujifilm Wako Pure Chemical Industry Co., Osaka, Japan) were used without purification. HPC (SSL grade, Nippon Soda Co., Ltd., Tokyo, Japan) was meshed by 425 μm after drying. PVP (Nakarai Tesque Inc., (K30), Kyoto, Japan) was used after drying.

### 3.2. Methods

#### 3.2.1. Sample Preparations

The mixed sample of 0.5 g of RSP at a mass ratio of 1:1:5 (RB, SDS, and PVP) was prepared. Another mixture, RSH, at a mass ratio of 1:1:3 (RB, SDS, and HPC-SSL) was prepared.

PM samples were prepared by vortexing (VORTEX-GENIE 2, Scientific Industries, Inc., Bohemia, NY, USA) for 2 min at frequency level 8. GM samples were prepared by mechanochemical synthesis with ball milling. The ball milling (MM400, Verder Scientific Co., Ltd., Tokyo, Japan) conditions were as follows; 0.5 g sample weight, 10 mL stainless pod with a stainless ball of *D* = 12 mm, 30 Hz frequency, 30 min milling time (twice 15 min milling), 5 min liquid nitrogen cooling.

The humidity-controlled desiccator in the convection oven at 40 °C (FC-610, Advantec Co., Tokyo, Japan) was applied to store the RB samples at an RH of 30% (saturated magnesium chloride solution) and 75% (saturated sodium chloride solution). The samples were stored in the desiccator in a vial bottle using KimWipes (Kimberly Clark Corp., Irving, TX, USA) as a breathable lid for 4 weeks. The prepared samples were evaluated using PXRD analysis, NIR spectroscopy, and ATR-MIR spectroscopy.

#### 3.2.2. PXRD

The prepared samples were analyzed with a PXRD diffractometer (RINT-2000, Rigaku Co. Ltd., Tokyo, Japan) at room temperature. Diffractogram measurements were as follows; 40 kV, 40 mA, Ni-filtered Cu Kα radiation, 0.02° step slit, 2*θ* = 5.00–30.00° measurement range. The powder was pre-ground to prevent crystal orientation and loosely packed in the glass holder.

#### 3.2.3. NIR Spectroscopy

NIR spectra of prepared samples in the glass bottles were measured using DLP NIR scan™ NIR-S-G1 (InnoSpectra Co., Hsinchu, Taiwan) at room temperature. The spectra measurement conditions of the samples were observed using the reflectance measurement mode. The reference sample was selected as a standard white plate (073G (6916-H422A), JASCO Co., Tokyo, Japan). The wavelength was in the range of 900 to 1700 nm with a resolution of 4 nm. NIR spectra were evaluated in the range of 900 to 1600 nm.

#### 3.2.4. ATR-MIR Spectroscopy

MIR spectra of samples were measured with a Fourier transform (FT)-IR spectrometer (Perkin Elmer, Norwalk, MA, USA) equipped with an ATR accessory. The ATR attachment was inserted directly in the light beam. The prepared sample powder was placed on the diamond of the ATR accessory at room temperature. ATR-IR spectra of the suspensions were measured at a range of 4000 to 400 cm^−1^ and a resolution of 1 cm^−1^. The spectra obtained were averaged and corrected for air background. The ranges 4000 to 2500 cm^−1^ and 2000 to 1000 cm^−1^ were evaluated.

#### 3.2.5. Simulated Vibrational Frequencies

The density functional method was applied to investigate IR vibrational modes of an RB molecule. An RB molecule was optimized in a vacuum based on B3LYP functions. The calculations of vibrational frequencies were obtained from the Hessian matrix. Convergence tolerance on both calculations was 0.001 Ha. The calculation details were described previously [60,61,62].

#### 3.2.6. PCA for Multiple Datasets

Multiple datasets of NIR spectra and PXRD patterns of the prepared samples were constructed via unit vector normalization. The resolution of degree on obtained PXRD patterns was compressed 0.08 degrees to reduce the time of PCA model construction. The dataset was evaluated using the variation by loadings and PCA scores on a singular value decomposition algorithm. The details of PCA and the algorithm were described previously [58].

PCA was fitted by obtaining eigenvalues of the variance-covariance matrix of the origin of the data set and the eigenvectors. The mean-centered data matrix *X* (NIR-PXRD dataset) was reduced to the sum of a cross product of two smaller matrices that were *P* and *T*, and a residual matrix *E* (Equation (1)).

*X* = *TP^T^* + *E*, (1)

where *T* is the matrix of the eigenvectors (loadings), and *P* is the matrix of scores. The cross product of *TP^T^* contains most of the original variance in *X*. This term is the structured part of the data; the most informative part. *E* represents the fraction of variation that cannot be modeled well [63].

#### 3.2.7. Software

The molecular orbital calculation was carried out using BIOVIA Materials studio 2019 (ver. 19.1.0.2353) with DMol^3^ package [64]. The chemometrics was performed using The Unscrambler^®^ X version 10.5.1 (64 bit) from CAMO Software (Computer-Aided Modeling, Trondheim, Norway) [65].

#### 3.2.8. DVS Measurements

Vapor sorption isotherm was observed by DVS at 25 °C using a Dynamic Vapor Sorption Advantage instrument (SMS Ltd., London, UK). The samples were mounted on a balance, and the RH was increased from 0 to 80%, 5% stepwise. The waiting time for 0.002% weight change was set to be 10 min, and the further steps were controlled automatically.

## 4. Conclusions

The effect of humidity-controlled storage on amorphous RB, RB form I and RB solid dispersion with different surfactant and polymers was investigated. Stored amorphous RB samples were transformed into RB form I after 1 week in RH 75%. Absorbance peaks due to amorphous RB was detected at 1505 nm. The IR spectra of RB samples were compared with simulated IR spectra of RB molecule using the molecular orbital method. Hydrogen bond points of RB indicated three points in the molecule, and the IR peak broadening suggested hydrogen bond network transformations.

The effect of PVP and HPC on solid dispersion RB and SDS samples were also investigated. Solid dispersion mixture of RB, SDS, and PVP, showed phase transformation into a hard, glassy sample. It was found that RB in the HPC-SDS solid dispersion had an amorphous halo, mostly amorphous. The contents of HPC and SDS in the solid dispersion inhibited RB recrystallization in RH 75%.

Combined NIR and PXRD dataset were applied to PCA of multivariate analysis. It was found that the combined data could evaluate correlation points between NIR spectra and diffraction patterns in both neat RB and solid dispersion samples. The PCA enabled the separation crystallinity cluster from scores evaluation. PCA based on multi-spectra dataset suggested a correlation among intermolecular interaction, NIR spectra, and PXRD patterns of the RB solid dispersion samples. Solid dispersion sample that could not be obtained with a single measurement data was evaluated using multispectral PCA. In a further study, we would like to report predictive evaluation of the physical properties of amorphous API samples from a multi-spectra dataset.

## Figures and Tables

**Table 1:** Correlation values among amorphous RB form I and prepared RB samples.

MIR Spectra *R*^2^ Values	RB Form I		Amorphous RB	
	RH 30%	RH 75%	RH 30%	RH 75%
RB form I	0.990	0.992	0.846	0.981
Amorphous RB	0.774	0.808	0.920	0.836

RH; relative humidity. RB; rebamipide. MIR; mid-infrared.

**Table 2:** Cumulative percentage of variances of PCA based on NIR-PXRD multi-dataset.

Component	Amorphous and RB form I			RSP Samples			RSH Samples		
PC	Cal.	Val.	Dif.	Cal.	Val.	Dif.	Cal.	Val.	Dif.
PC1	88.8	87.8	87.8	77.86	74.78	74.78	83.9	81.9	81.9
PC2	95.2	93.8	6.0	92.11	83.76	8.98	93.6	91.8	9.9
PC3	96.3	94.4	0.6	94.95	83.87	0.11	95.9	93.7	1.9
PC4	96.9	94.8	0.5	96.71	85.40	1.53	96.9	94.2	0.5
PC5	97.3	94.9	0.02	97.66	86.17	0.77	97.6	94.6	0.4

Cal; calibration model. Val; validation modes. Dif; cumulative percentage variance difference between one lower component model and the models on validation models. RB; rebamipide. NIR; near-infrared. PCA; principal component analysis.

## Supplementary Materials

The following are available online at https://www.mdpi.com/1424-8247/13/7/147/s1, Figure S1: Images of bulk materials, Figure S2: Images of RB-SDS-PVP (RSP) mixture samples; Figure S3: Simulated infrared (IR) spectra based on DFT calculation; Figure S4: Images of RB-SDS-HPC (RSH) mixture samples; Table S1: Simulated IR spectra intensities.
