# Supplementary material for: Effect of Polymers and Storage Relative Humidity on Amorphous Rebamipide and Its Solid Dispersion Transformation: Multiple Spectra Chemometrics of Powder X-Ray Diffraction and Near-Infrared Spectroscopy
Source: Pharmaceuticals (Basel). 2020 Jul 10;13(7):147. doi: 10.3390/ph13070147 (PMC7407760; doi:10.3390/ph13070147)
Supplement: Supplementary file 1 [file pharmaceuticals-13-00147-s001.pdf]

Effect of Polymers and Storage Relative Humidity on Amorphous Rebamipide and Its Solid Dispersion Transformation: Multiple Spectra Chemometrics of Powder X-ray Diffraction and Near-Infrared Spectroscopy

Yuta Otsuka [1,2,†], Yuiko Utsunomiya [1], Daiki Umeda [3], Etsuo Yonemochi [3], Yayoi Kawano [1,2,†] and Takehisa Hanawa [1,2,*]

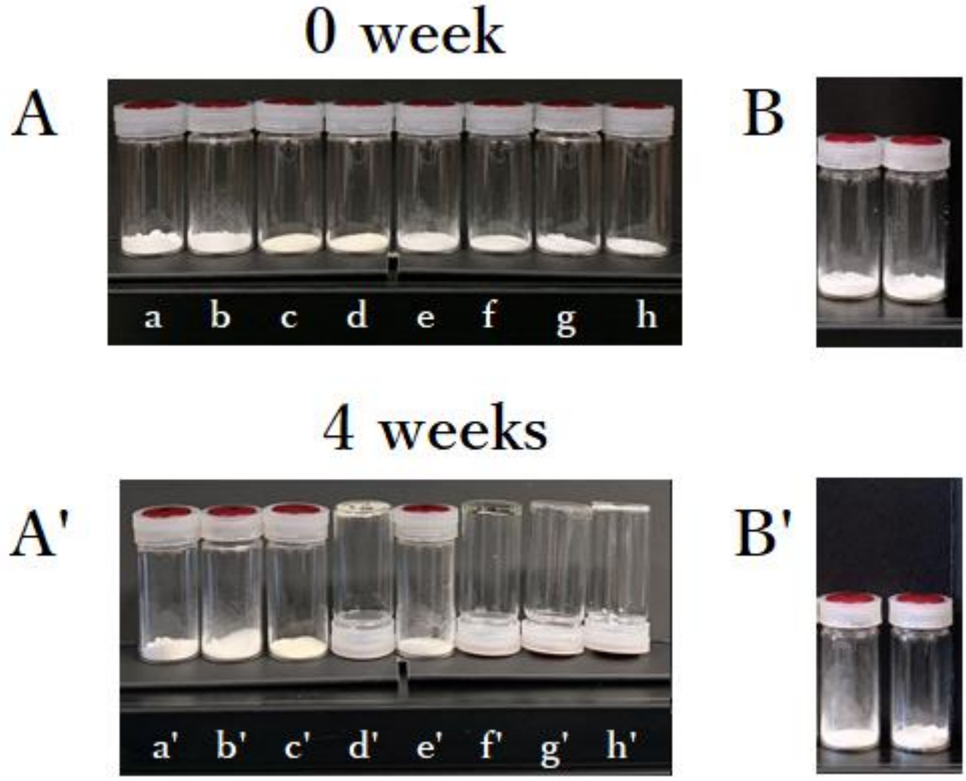

Figure S1. Photographic images of bulk materials (**A**) and RB samples (**B**). The upper figure shows the 0-week sample and the samples of lower shows after stored 4-week samples. RB form I stored in RH 30% (**a**) and RH 75% (**b**); HPC-SSL sample stored in RH 30% (**c**) and RH 75% (**d**); PVP sample stored in RH 30% (**e**) and RH 75% (**f**); SDS sample stored in RH 30% (**g**) and RH 75% (**h**). Ground RB stored in RH 30% and RH 75%.

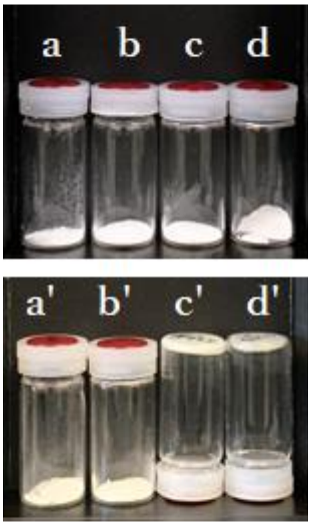

Figure S2. Photographic images of RB-SDS-PVP mixture samples. Upper figure (**a**–**d**) shows 0-week samples, and the lower figure (**a'**–**d'**) shows samples after 4-week storage. RSP PM sample RH 30% (**a**) and RH 75% (**b**); RSP GM sample RH 30% (**c**) and RH 75% (**d**).

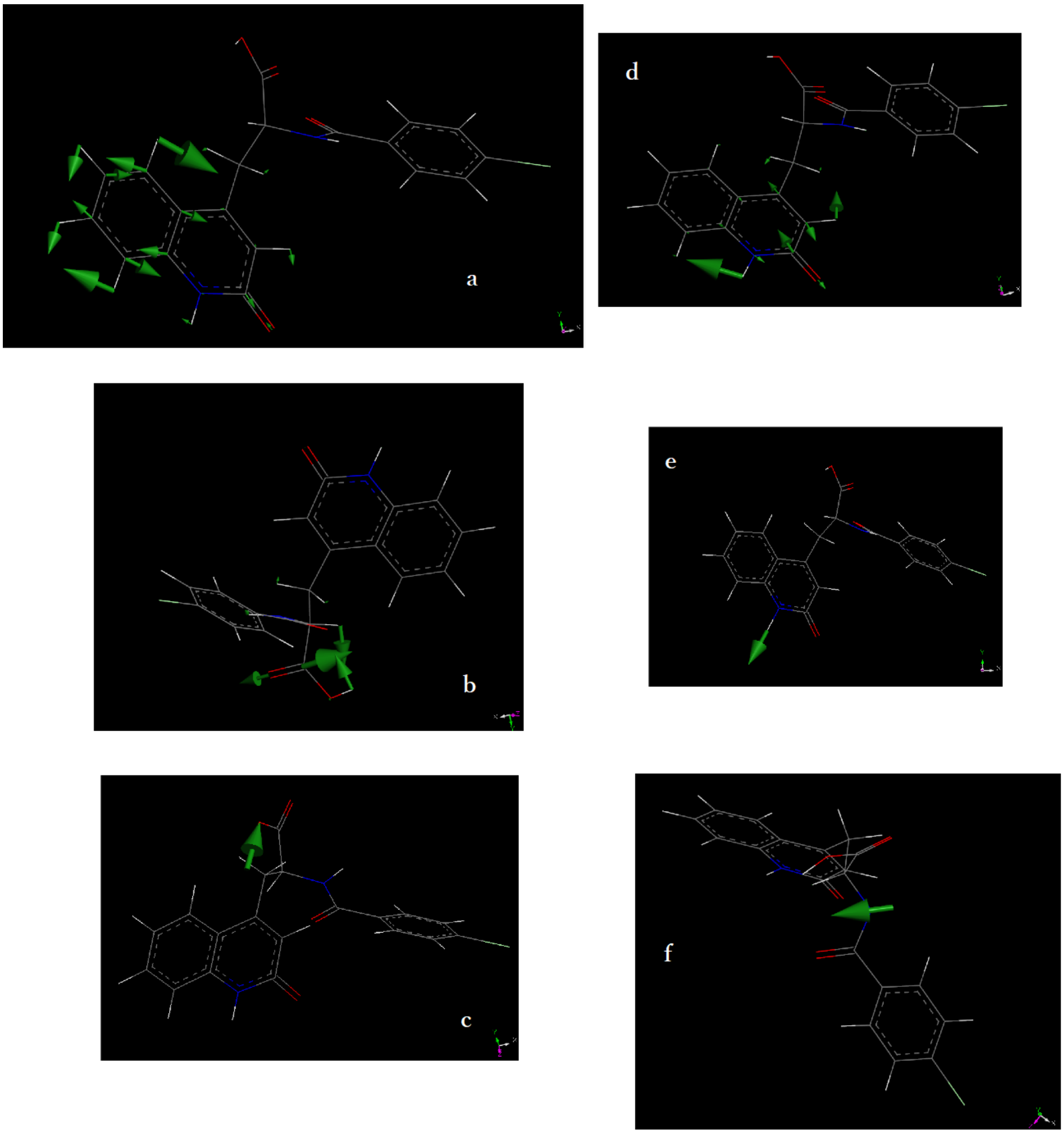

Figure S3. Simulated IR spectra based on DFT calculation; 1653 cm^{-1} of IR vibration (Figure S3a); 1687 cm^{-1} of IR vibration (Figure S3b); 1734 cm^{-1} of IR vibration (Figure S3c); 3566 cm^{-1} of IR vibration (Figure S3d); 3594 cm^{-1} of IR vibration (Figure S3e); 3606 cm^{-1} of IR vibration (Figure S3f).

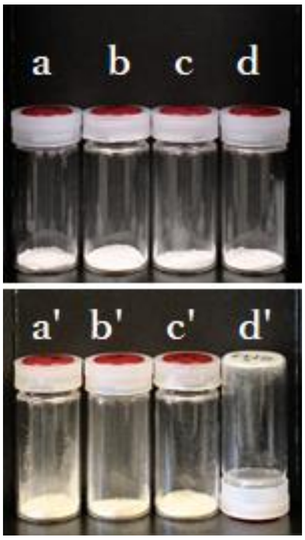

Figure S4. Photographic images of RB-SDS-HPC mixture samples. Upper figure (**a**–**d**) shows 0-week samples, and the lower figure (**a**′–**d**′) shows samples after 4-week storage. RSH PM sample RH 30% (**a**) and RH 75% (**b**); RSH GM sample RH 30% (**c**) and RH 75% (**d**).

Table S1. Simulated IR spectra intensities; A RB molecule were optimized in vacuum based on B3LYP functions.

Function	B3LYP
Frequency (1/cm)	Intensity (km/mol)
-58.1	8.4
25.7	0.6
37.9	1.5
40.7	2.8
55.0	3.1
68.8	2.2
76.6	5.0
91.8	2.1
113.6	3.8
117.8	4.0
139.6	1.6
178.2	1.0
192.7	7.1
212.5	8.3
256.7	8.4
269.1	7.3
283.3	0.2
303.6	12.7
321.4	3.9
323.1	5.1
349.4	33.4
382.0	6.3
413.3	0.8
443.0	6.4
443.7	58.9
464.7	10.0
471.7	90.3
495.4	19.5
505.5	16.1
509.5	5.1

540.0	8.6
560.2	9.2
565.7	0.8
580.8	3.2
627.4	150.5
644.5	0.7
668.9	13.2
672.4	1.4
680.9	8.0
702.9	13.6
716.4	44.2
742.6	2.6
746.5	3.3
770.4	1.6
771.8	2.4
792.8	38.1
799.6	154.1
816.4	33.2
856.1	19.6
863.6	1.8
893.2	30.0
900.3	9.8
903.6	21.7
906.5	5.0
927.5	45.9
959.2	32.5
976.9	1.4
991.1	6.3
1013	0.1
1031	2.3
1034	0.4
1047	13.7
1059	44.6
1068	3.9

1069	7.9
1086	9.5
1103	33.2
1119	7.4
1146	109.4
1153	15.9
1181	379.8
1187	4.4
1223	4.2
1232	12.4
1247	7.5
1256	12.2
1286	16.8
1302	86.0
1303	176.6
1327	6.4
1334	46.5
1359	14.1
1366	20.1
1370	0.9
1381	32.2
1423	48.6
1432	63.6
1442	6.1
1455	20.6
1502	15.7
1515	313.0
1543	22.2
1547	18.3
1557	14.5
1586	16.5
1594	96.4
1631	25.3
1632	161.1

1634	57.4
1653	8.0
1687	582.9
1735	219.3
3047	12.4
3072	4.0
3094	9.9
3169	5.1
3176	8.8
3180	2.4
3196	20.7
3198	0.5
3205	14.8
3205	3.7
3213	0.7
3216	0.9
3567	51.8
3595	50.1
3607	36.0
